# DynaMut2: Assessing changes in stability and flexibility upon single and multiple point missense mutations

**DOI:** 10.1002/pro.3942

**Published:** 2020-09-11

**Authors:** Carlos H.M. Rodrigues, Douglas E.V. Pires, David B. Ascher

**Affiliations:** ^1^ Structural Biology and Bioinformatics, Department of Biochemistry, Bio21 Institute University of Melbourne Melbourne Victoria Australia; ^2^ Computational Biology and Clinical Informatics Baker Heart and Diabetes Institute Melbourne Victoria Australia; ^3^ School of Computing and Information Systems University of Melbourne Melbourne Victoria Australia; ^4^ Department of Biochemistry University of Cambridge Cambridge UK

**Keywords:** dynamics, graph‐based signatures, missense mutations, stability changes

## Abstract

Predicting the effect of missense variations on protein stability and dynamics is important for understanding their role in diseases, and the link between protein structure and function. Approaches to estimate these changes have been proposed, but most only consider single‐point missense variants and a static state of the protein, with those that incorporate dynamics are computationally expensive. Here we present DynaMut2, a web server that combines Normal Mode Analysis (NMA) methods to capture protein motion and our graph‐based signatures to represent the wildtype environment to investigate the effects of single and multiple point mutations on protein stability and dynamics. DynaMut2 was able to accurately predict the effects of missense mutations on protein stability, achieving Pearson's correlation of up to 0.72 (RMSE: 1.02 kcal/mol) on a single point and 0.64 (RMSE: 1.80 kcal/mol) on multiple‐point missense mutations across 10‐fold cross‐validation and independent blind tests. For single‐point mutations, DynaMut2 achieved comparable performance with other methods when predicting variations in Gibbs Free Energy (ΔΔ*G*) and in melting temperature (Δ*T*_m_). We anticipate our tool to be a valuable suite for the study of protein flexibility analysis and the study of the role of variants in disease. DynaMut2 is freely available as a web server and API at http://biosig.unimelb.edu.au/dynamut2.

## 1 INTRODUCTION

Proteins are highly dynamic, metastable molecular machines. Missense mutations are associated with more than half of all known inherited diseases, however, they are often associated with more subtle molecular effects than mutations that lead to larger changes to the mature peptide. These single amino acid changes can readily disrupt the intricate network of intramolecular interactions, affecting how a protein folds, its stability, dynamics, and ultimately protein function. Beyond phenotypic outcomes,^1^, ^2^, ^3^, ^4^, ^5^, ^6^, ^7^, ^8^, ^9^, ^10^, ^11^, ^12^, ^13^, ^14^, ^15^, ^16^, ^17^, ^18^, ^19^, ^20^, ^21^, ^22^ it also has direct implications for their experimental study, protein engineering,^23^, ^24^ drug design,^25^, ^26^, ^27^, ^28^, ^29^, ^30^ and use in industrial processes.^31^

A number of approaches have been developed to predict how missense mutations affect protein stability using either sequence^32^, ^33^, ^34^ or structural information.^35^, ^36^, ^37^ The information from both approaches is often complementary; however, structural methods have generally assumed a protein is static and does not consider the implications of a mutation within its conformational landscape. We previously showed that by considering both the mutation environment and the protein dynamics, we could more accurately predict the effects of single‐point missense mutations.^38^

Most predictive tools, however, have been limited to single point missense variants, and the inclusion of protein dynamics computationally scales poorly with protein size. Here we present DynaMut2, an enhanced server that combines normal mode analysis with our graph‐based representation of protein structure, to accurately and quickly predict the effects of single and multiple point mutations on protein stability and dynamics.

## 2 RESULTS AND DISCUSSION

**FIGURE 1:**
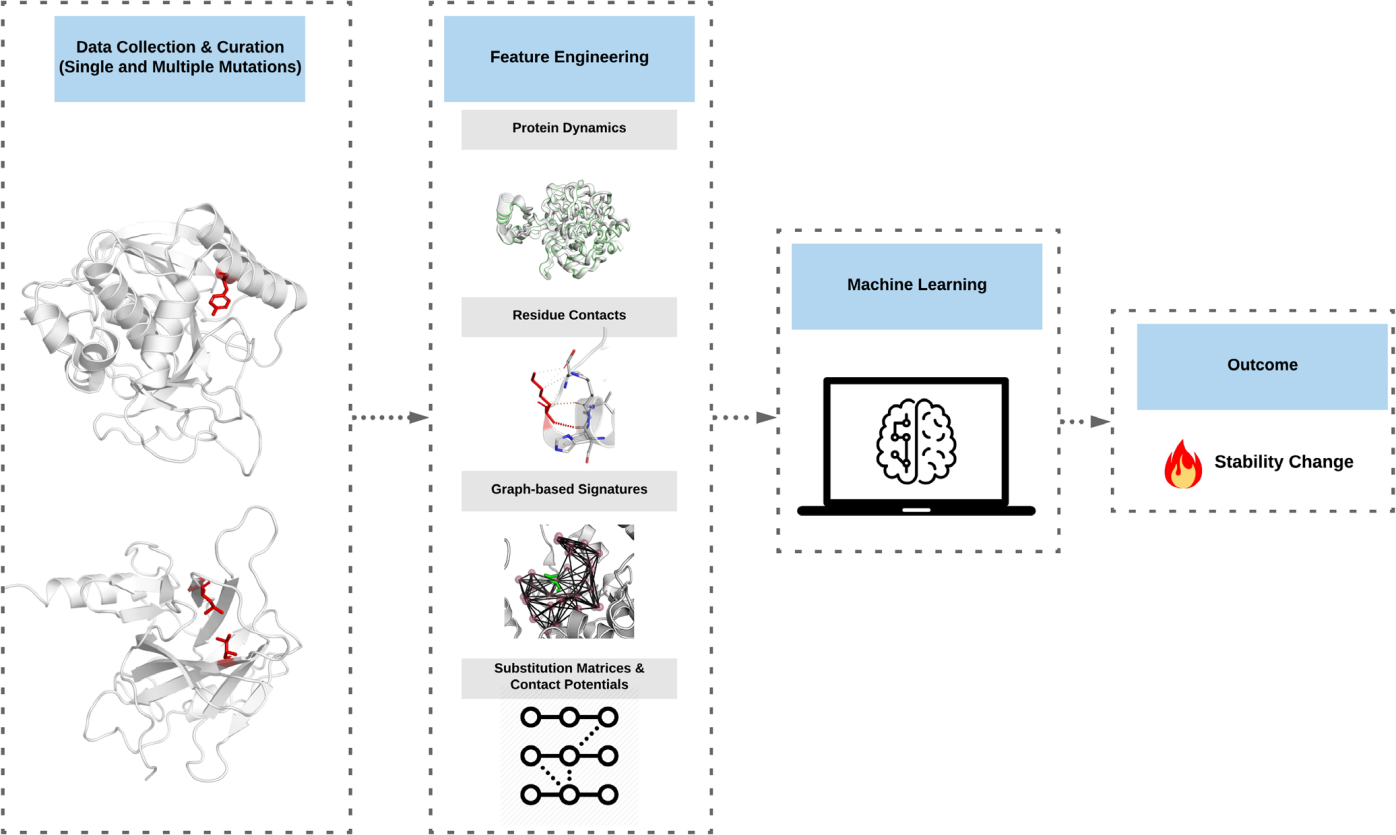
DynaMut2 workflow. The methodology for this work can be summarized into four steps: (1) data collection and curation of single and multiple mutations, (2) feature engineering to model the effects of mutations, (3) supervised machine learning, and (4) the predicted effects on stability and dynamics

The DynaMut2 development workflow is summarized in Figure 1. Data on single and multiple point mutations were derived from ProTherm.^39^ Given the wide range of molecular mechanisms by which mutations can impact protein function, we modeled the effects of each mutation using a range of features, including protein dynamics (NMA), wild‐type residue environment, substitution propensities and contact potential scores, interatomic interactions^40^ and also our well‐validated graph‐based signatures approach.^35^, ^41^, ^42^, ^43^, ^44^, ^45^, ^46^, ^47^, ^48^ These were then used to train and test machine learning algorithms. Our predictive models were further evaluated using independent blind test sets.

### 2.1 *Predicting the effects of single point mutations*

**FIGURE 2:**
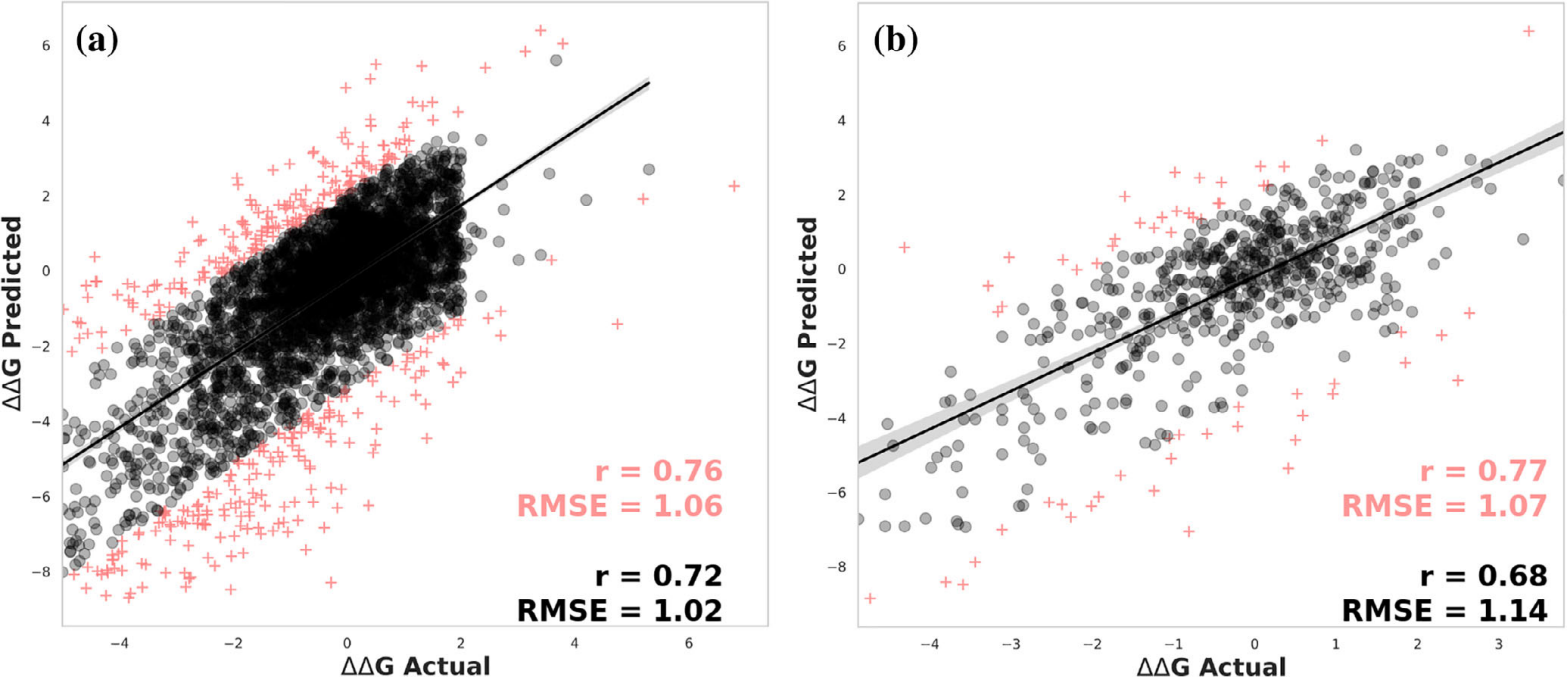
Predictive performance of DynaMut2 on 10‐fold cross‐validation (a) and non‐redundant test sets (b) for single point mutations. 10% of outliers are shown as pink crosses

We initially evaluated the performance of our approach to predict changes in stability caused by single point mutations. DynaMut2 was able to achieve a Pearson's correlation of *r* = 0.72 (RMSE = 1.02 kcal/mol) for the dataset S4022, under 10‐fold cross‐validation, and *r* = 0.68 on S611, our non‐redundant independent test set (RMSE = 1.14 kcal/mol) (Figure 2), outperforming all other methods (Table 1). The comparable performance between cross‐validation and non‐redundant blind test supports the generalizability of the final model. After removing 10% of outliers, performance remained consistent for the training set with *r* = 0.76 (RMSE = 1.06), and increased to *r* = 0.77 (RMSE = 1.07) on the test set. No significant differences in the distributions of properties were observed for the outliers compared to the overall dataset.

**TABLE 1:** Comparative performance across the non‐redundant test set S611

Method	Overall		Stabilizing mutations		Destabilizing mutations		AUC
	Pearson (*r*)	RMSE (kcal/Mol)	Pearson (*r*)	RMSE (kcal/Mol)	Pearson (*r*)	RMSE (kcal/Mol)	
DynaMut2	**0.68**	**1.14**	**0.51**	**1.02**	**0.62**	**0.91**	**0.68**
DynaMut1	0.49^*^	1.38^+^	0.47	1.24	0.55	1.01	0.62
SDM	0.35^*^	1.93^+^	0.15^*^	2.00^+^	0.36^*^	1.86^+^	0.60^#^
mCSM	0.46^*^	1.42^+^	0.11^*^	1.81^+^	0.56	0.98	0.56^#^
DUET	0.48^*^	1.40^+^	0.09^*^	1.75^+^	0.58	1.00	0.56^#^
ENCoM	−0.14^*^	2.03^+^	−0.01^*^	1.94^+^	−0.18^*^	2.09^+^	0.41^#^
Maestro	−0.36^*^	1.55^+^	0.27^*^	1.17	0.43^*^	1.81^+^	0.46^#^
I‐mutant	0.33^*^	1.47^+^	0.03^*^	1.83^+^	0.49^*^	1.09^+^	0.51^#^
MUpro^a^	0.15^*^	1.71^+^	−0.05^*^	2.15^+^	0.23^*^	1.21^+^	0.50^#^

* *p* Value < .05 compared with DynaMut2 using z test.

# *p* Value < .05 compared with DynaMut2 using *t* test.

+ *p* Value < .05 compared with DynaMut2 using Diebold‐Mariano test.

^a^ 48 mutations were left out due to input issues.

Due to the natural imbalance between stabilizing and destabilizing mutations present in the training and evaluation data (Figure S1), we further analyzed performance on the respective classes separately. Across the non‐redundant validation se S611, DynaMut2 achieved a Pearson's correlation of *r* = 0.62 (RMSE = 1.75) and *r* = 0.51 (RMSE = 1.88) on destabilizing and stabilizing mutations, respectively. The slightly lower performance toward stabilizing mutations was expected due to the imbalanced distribution of data but was significantly improved compared to previous methods (Table 1). These results remained consistent when we compared the performances using rank coefficient scores Kendall and Spearman (Table S1). This was further reflected in the ability of DynaMut2 to correctly classify stabilizing and destabilizing mutations (AUC 0.68), outperforming previous approaches.

To further investigate potential biases in the predictive performance, we evaluated the performance of DynaMut2 on subsets derived from the O2567 dataset.^49^ DynaMut2 showed significantly better performance than all other approaches for mutations on buried residues (RSA ≤30%; Table S2). A small deterioration in performance is observed on mutations on exposed residues (RSA > 30%; Table S3), likely to be related to the smaller number features captured by the graph‐based signatures in DynaMut2; however, our method still achieved comparable results to mCSM, MAESTRO and SDM, and outperformed other approaches. Evaluating the performance on different protein CATH classifications, DynaMut2 outperformed other approaches across β‐sheet structures (Table S4), and α‐helix and β‐helix structures (Table S5). The size of the protein being mutated did not affect performance, with comparable performance between larger proteins (>150 residues; Table S6) and small proteins (<150 residues; Table S7), outperforming all other evaluated approaches. Similarly, DynaMut2 performance was similar to mutations from large to small residues (Table S8), from small to large residues (Table S9), or for mutations between residues of comparable sizes (Table S10). Encouragingly, DynaMut2 outperformed all other approaches on mutations leading to a change in volume and demonstrated comparable performance to the top approaches for mutations between residues of similar volume. Overall, this highlighted that DynaMut2 predictive performance across all single‐point mutations was significantly more balanced and less biased than all other methods evaluated.

We further evaluated the performance of our model across an additional independent test set, S276. DynaMut2 achieved a Pearson's correlation of 0.52, comparable with the best‐performing methods (Table 2) and significantly better than MUpro.^50^ Although not directly comparable, as there is a correlation between changes upon mutation in stability (Δ*G*) and thermal stability (*T*_m_),^51^ the performance of DynaMut2 on predicting changes in melting temperature was assessed using the blind test set S173. Results were stratified by protein structure and summarized in Table S11. Overall, DynaMut2 ranks fourth among the methods evaluated; however, performances of all methods varied greatly between structures. These results indicate a possible challenge in accurately predicting the thermal stability effects of mutations on a more diverse set of proteins.

**TABLE 2:** Comparative performance across the S276 blind test of experimental ΔΔ*G*

Method	*R*	MAE (kcal/Mol)
DynaMut2	0.52	0.88
DeepDDG	0.55	0.86
SDM	0.48	1.02
mCSM	0.46	0.90
I‐mutant	0.45	0.91
STRUM	0.44	0.88
MUpro	0.19*	1.06

* *p* Value < .05 compared with DynaMut2 using *z* test.

### 2.2 *Predicting the effects of multiple point mutations*

**FIGURE 3:**
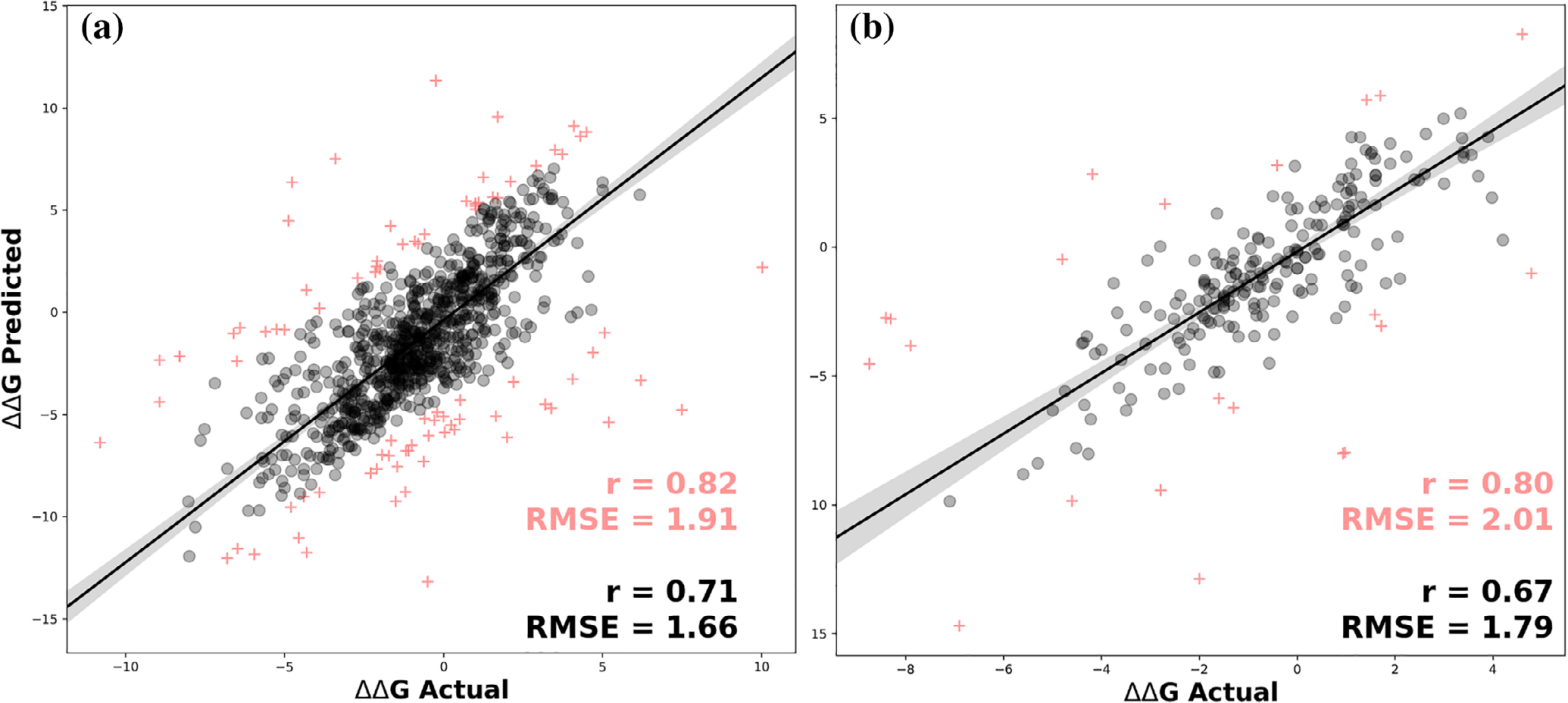
Predictive performance of DynaMut2 on 10‐fold cross‐validation (a) and non‐redundant test sets (b) for multiple point mutations. 10% of outliers are shown as pink crosses

The performance of our approach to predict the effects of multiple point mutations on protein stability was then assessed. DynaMut2 achieved a Pearson's correlation of *r* = 0.71 (RMSE = 1.66 kcal/mol) under 10‐fold cross‐validation and *r* = 0.67 (RMSE = 1.79 kcal/mol) on our non‐redundant test set. The comparable performance between cross‐validation and blind test set again gave confidence in the generalizability of the approach. This significantly outperformed the previously reported performances of DDGun, DDGun3D, Maestro, and FoldX, whose correlations ranged from 0.37 to 0.55 on the experimental multiple point mutations in ProTherm.^52^ Performances were consistent when considering only 90% of the data, with DynaMut2 achieving *r* = 0.82 (RMSE = 1.91) and *r* = 0.80 (RMSE = 2.01) on 10‐fold cross‐validation and blind‐test, respectively (Figure 3). This indicates that outlier predictions were not having a significant effect on the correlations.

The unbalanced nature of the training dataset was evident when we analyzed the performance of our final model on stabilizing and destabilizing multiple mutations separately (Table 3). Overall, DynaMut2 was able to correctly classify 80% of multiple missense mutations (AUC 0.84) in the blind test set, including 93% of the destabilizing mutations, providing confidence in the ranking ability of the approach. As expected, however, across our non‐redundant test set DynaMut2 shows a better performance toward predicting multiple mutations with a destabilizing effect, achieving a Pearson's correlation of *r* = 0.56 (RMSE = 2.66), while for stabilizing entries the performance drops to *r* = 0.42 (RMSE = 2.94). These results indicate a need for new experimental data on multiple point mutations, especially those with stabilizing effects, since the use of hypothetical reverse mutations is likely to add more uncertainty to the model.

**TABLE 3:** Comparative performance on multiple mutations prediction across different correlation coefficients

Methods	Overall			Stabilizing			Destabilizing		
	*r*_p_	Tau	*r*_s_	*r*_p_	Tau	*r*_s_	*r*_p_	Tau	*r*_s_
**DynaMut2**	**0.71**	**0.58**	**0.75**	**0.42**	**0.38**	**0.53**	**0.56**	**0.47**	**0.63**
MAESTRO	0.19^*^	0.13^+^	0.19^#^	0.12^*^	0.07^+^	0.08^#^	0.21^*^	0.14^+^	0.21^#^
FoldX	0.33^*^	0.21^+^	0.31^#^	0.04^*^	0.06^+^	0.09^#^	0.30^*^	0.19^+^	0.27^#^

* *p* Value < .05 compared with DynaMut2 using Fisher r‐to‐z transformation.

+ *p* Value <.05 by transforming tau‐to‐r followed by Fisher r‐to‐z transformation.

# *p* Value <.05 by transforming rho‐to‐r followed by Fisher r‐to‐z transformation.

### 2.3 *Web server*

We have implemented DynaMut2 as a freely available and user‐friendly web server (http://biosig.unimelb.edu.au/dynamut2/). The frontend was developed using Materializecss version 1.0.0 and the backend uses the Flask module (1.0.2) from the Python programming language. The web server is hosted on a Linux machine running Apache.

### 2.4 *Input*

DynaMut2 can be used in three different ways^1^: predicting ΔΔ*G* for single point mutations,^2^ predicting ΔΔ*G* for multiple point mutations (up to three), and also analysis of protein dynamics based on NMA. For predicting single point mutations, similarly to our previous implementation of DynaMut, two different inputs are available: “Single mutation” and “List of Mutations”. For the Single Mutation option, users are required to provide a protein structure on PDB format or provide a four‐digit code of an entry on the PDB, the chain identifier where the mutation occurs and the point mutation defined as string comprising wild‐type residue one‐letter code, residue position, and mutant residue one‐letter code. For the List of Mutations option, users must provide the structure of the protein, similarly to the Single Mutation option, and also upload a file with the list of variants (one per line), following the same mutation code previously defined.

For predicting the effects of multiple mutations, users are required to provide the structure of the protein, as previously described, and also the multiple mutations separated by a comma. DynaMut2 also allows for submitting a list of multiple point mutations to be analyzed in batch. These can be input by uploading a file with one entry of multiple mutations separated by comma per line.

Alternatively, for protein dynamic analysis, users are required to input the protein structure by uploading a file using the PDB format or provide a valid four‐digit code for a PDB entry, and also select one of the force fields available to guide structural interactions for NMA. All force field options available are detailed in Table S12.

### 2.5 *Output*

**FIGURE 4:**
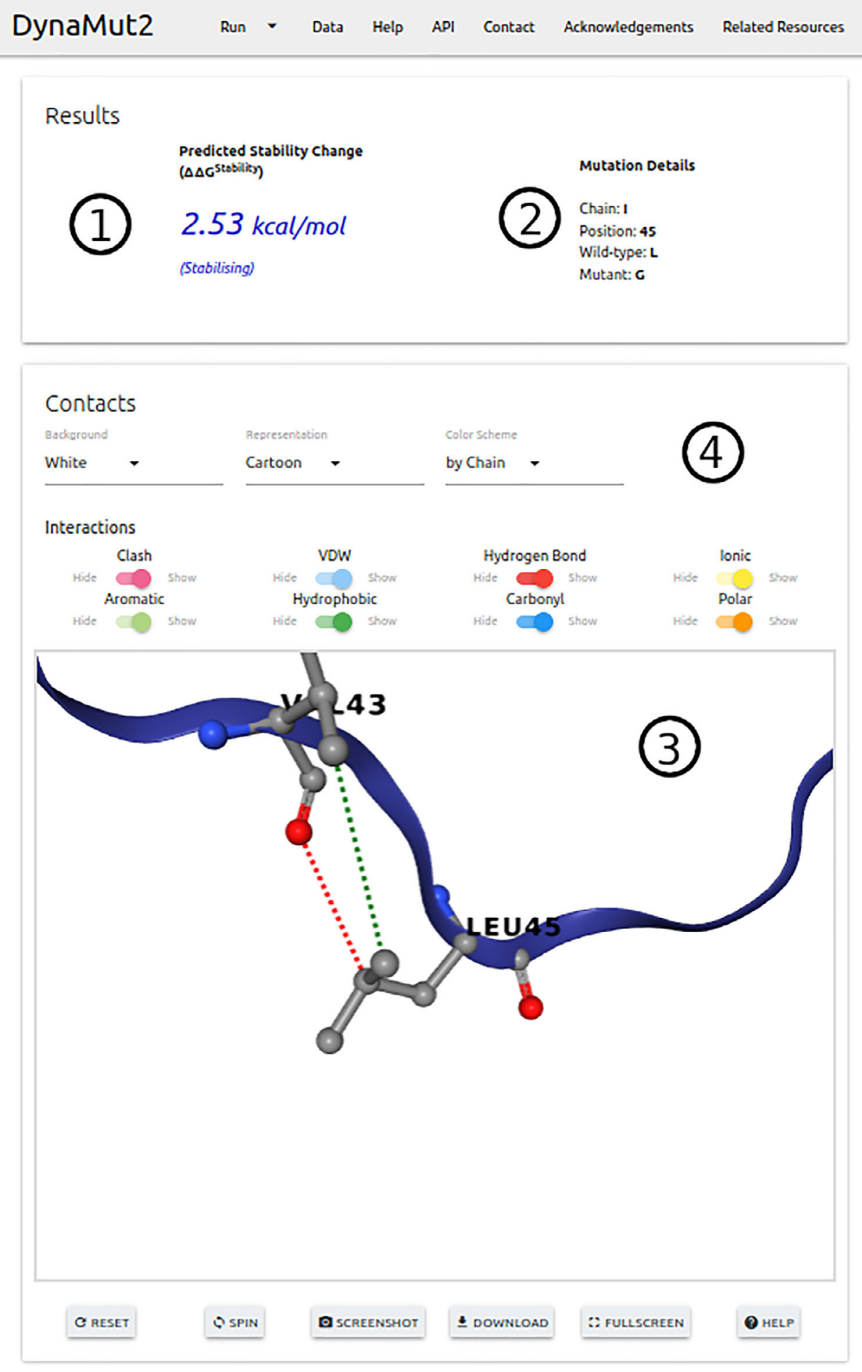
DynaMut2 results page. The figure depicts the prediction results page for single‐point mutations. The predicted effect of a mutation in stability and dynamics is given as the variation in Gibbs Free Energy (in Kcal/mol) (1), together with complementary information about the mutation provided (2). Users can inspect the wild‐type residue environment via an interactive viewer (3), which also allows the visualization of non‐covalent interactions established by the mutated residue

For single point mutations on the “Single Mutation” option, predicted ΔΔ*G* is shown at the top with details of users' input and also the wild‐type residue environment (Figure 4). All interatomic contacts calculated with Arpeggio are also displayed as an interactive viewer using NGL viewer.^53^ On the “Mutation List” option, the results are displayed as a downloadable table with options to view details of each variation separately, similarly to the analysis provided by the “Single Mutation”, option.

The results for multiple mutations, predictions are displayed at the top of the page with detailed information for each mutation, if a list is provided these results are shown as a table. An interactive viewer allowing for the analysis of residue contacts is also available.

For NMA submissions, the results are displayed on three panels. The first two provide information on trajectory representation of the molecule motion and porcupine plots, summarizing vector field representation, for the first non‐trivial modes. Finally, the last panel displays a residue correlation matrix and structural representations using all modes.

### 2.6 *API*

DynaMut2 conveniently offers an API (Application Programming Interface) to assist users in integrating our predictive tool into their research pipelines. All jobs submitted to DynaMut2 are labeled with a unique identifier, which is used to query the status of the job. Input fields follow the same rules of our website implementation. A full description of these with examples using curl and Python are available at http://biosig.unimelb.edu.au/dynamut2/api.

### 2.7 *Processing time*

Finally, we compared the performance of DynaMut2 with our previous implementation, DynaMut, in terms of processing time for single‐point mutations on six different protein structures. For each structure, we submitted a single‐point mutation to each server and computed the processing time in seconds. This procedure was repeated 10 times for each mutation and the results are summarized in Table S13 and Figure S2. Clearly, the greater the number of residues comprising the protein structure the longer it takes for our NMA based methods to generate predictions. However, DynaMut2 runs much faster than DynaMut on all sets of experiments with very little differences in each repetition and an improvement of more than six times in the worst‐case scenario.

## 3 CONCLUSION

Here we present DynaMut2, a tool that incorporates information on protein dynamics and structural environment properties of wild‐type residue with our graph‐based signatures approach to provide an accurate prediction of mutation effects on stability and dynamics for single and multiple point mutations. Our updated server has shown to outperform other methods on predicting changes in stability caused by single point mutations and also comparable results for when used for estimating Δ*T*_m_. In addition, our new approach was significantly faster compared to the original DynaMut, which will be of great benefit toward large scale analysis and large structures. Finally, we have extended our method to predict the effects of multiple point mutations (double and triple mutants) and an API, which conveniently enables users to programmatically run predictions and represents a great contribution in terms of a novelty for this type of tool. We believe DynaMut2 represents an invaluable resource for the study of protein dynamics and to help understand the role of mutations in diseases. Web server and API with examples are freely available at http://biosig.unimelb.edu.au/dynamut2.

## 4 MATERIALS AND METHODS

### 4.1 *Data set*

We have collected experimental data on 2,648 single point mutations on 125 globular proteins from Protherm.^39^ Of these, 2,080 are destabilizing (ΔΔ*G* < 0.0 kcal/mol) and 568 stabilizing (ΔΔ*G* > 0.0 kcal/mol) (Figure S1). To minimize the imbalanced nature of our dataset (Figure S1) and as a sanity check evidenced by other studies, here we use hypothetical reverse mutations.^36^, ^54^ However, differently from our previous implementation of DynaMut, hypothetical reverse mutations with more drastic changes on Gibbs free energy (ΔΔ*G* < −2.0 kcal/mol or ΔΔ*G* > 2.0 kcal/mol) were left out of our study due to uncertainties about the quality and biological significance of the modeled mutant. Our final dataset comprised 4,633 mutations (2,640 destabilizing and 1,993 stabilizing), which were split into 4,022 entries (S4022) for training our predictive model and a non‐redundant test set comprising 611 entries (S611), following the protocol from our initial version of DynaMut.^38^ For further performance evaluation and comparison with other methods, here we also consider a test set of 276 mutations (S276) with low sequence identity to proteins in the original ProTherm dataset, and an independent test set comprising 173 variants (S173) in six proteins with experimental melting temperature changes available (Δ*T*_m_). The latter includes the structure of guanylate kinase (GK) obtained through homology modeling with Modeller^55^ using the mouse GK as a template (PDB: 1LVG), similarly to previous works.^56^, ^57^

For the data on multiple point mutations, we were able to extract 1,323 entries from ProTherm; however, since the majority of entries were double and triple mutants (Figure S3) and for the sake of simplicity, here we only considered those two types. Our final dataset comprised 1,098 entries (710 destabilizing and 388 stabilizing) (Figure S4), which were randomly split into train and test sets comprising 872 and 227 entries, respectively.

In this study, we prioritize the use of biological assembly structures author assigned, if not available, for structures generated using NMR for instance, the asymmetric unit was considered. All data used in this study is freely available for download at http://biosig.unimelb.edu.au/dynamut2/data.

### 4.2 *Normal model analysis*

NMA provides a valuable approach for the study of dynamics and accessible conformations in a system as an alternative to time‐consuming and computationally expensive Molecular Dynamics simulations. Similarly with our previous work, here we incorporated dynamics properties extracted from the protein structure generated with the module NMA of the bio3D tool.^58^

### 4.3 *Graph‐based signatures*

Our in‐house graph‐based signatures approach to represent molecular structures^35^, ^59^, ^60^, ^61^ has proven to be successful for a range of applications toward the study of protein structure and changes carried out by missense mutations,^35^, ^37^, ^41^, ^42^, ^43^, ^44^, ^45^, ^46^, ^47^, ^48^ including phenotypic changes.^16^, ^62^, ^63^ These signatures comprise physicochemical and geometrical properties from the wild‐type environment based on distance patterns mined from the 3D structure by representing atoms as nodes and their interactions as edges. Physicochemical properties are then defined based upon the amino acid properties, namely pharmacophore, and distance patterns between atoms are summarized as cumulative distribution functions.

### 4.4 *Analysis of mutation effects*

Changes in Gibbs Free energy of folding can occur due to a myriad of factors related and in order to incorporate these properties, we used Arpeggio^40^ to calculate the number of hydrophobic contacts involving the wild‐type residue and contact potential scores from AAINDEX database.^64^

### 4.5 *Machine learning*

In this study, we used the implementation of the Random Forest algorithm available on the scikit‐learn Python library for both the prediction of ΔΔ*G* for single and multiple mutations. In order to avoid the curse of dimensionality and improve performance, we selected our features using an incremental stepwise greedy approach.

## 5 GENERAL STATEMENT

Small changes in proteins can have large phenotypic outcomes. By considering the changes of mutations within the context of the protein 3D structure, we have been able to accurately predict the molecular consequences of single and multiple point mutations on protein folding, stability, and dynamics. We have made this tool available through an easy to use website and API.

## AUTHOR CONTRIBUTIONS

**Carlos Rodrigues:** Data curation; formal analysis; investigation; methodology; validation; visualization; writing‐original draft. **Douglas Pires:** Formal analysis; methodology; writing‐review and editing. **David Ascher:** Conceptualization; data curation; formal analysis; funding acquisition; investigation; methodology; project administration; supervision; validation; writing‐original draft; writing‐review and editing.

## CONFLICT OF INTEREST

No conflict of interest declared.

## Supporting information


**Appendix** **S1**: Distribution of data used to train and evaluate the models (Figures S1, S3, S4). Comparison of prediction time between DynaMut and DynaMut2 (Figure S2 and Table S13). Comparison of rank coefficient scores for single point mutation predictions (Table S1). Performance across different classes of mutations from the O2567 non‐redundant test sets (Table S2–S10). Comparison of performance effects of single point mutations on Δ*T*_m_ (Table S11). Description of NMA forcefields available on DynaMut2 (Table S12).
