# Supplementary material for: DynaMut2: Assessing changes in stability and flexibility upon single and multiple point missense mutations
Source: Protein Sci. 2020 Sep 11;30(1):60–9. doi: 10.1002/pro.3942 (PMC7737773; doi:10.1002/pro.3942)
Supplement: Supplementary file 1 — Appendix S1: Distribution of data used to train and evaluate the models (Figures S1, S3, S4). Comparison of prediction time between DynaMut and DynaMut2 (Figure S2 and Table S13). Comparison of rank coefficient scores for single point mutation predictions (Table S1). Performance across different classes of mutations from the O2567 non‐redundant test sets (Table S2–S10). Comparison of performance effects of single point mutations on ΔT m (Table S11). Description of NMA forcefields available on DynaMut2 (Table S12). [file PRO-30-60-s001.docx]

**SUPPLEMENTARY MATERIAL**

**DynaMut2: Assessing changes in stability and flexibility upon single and multiple point missense mutations**

Carlos H.M. Rodrigues^1,2,*^, Douglas E.V. Pires^1,2,3,#^, David B. Ascher^1,2,4,#^

^1^Structural Biology and Bioinformatics, Department of Biochemistry, Bio21 Institute, University of Melbourne, Victoria, Australia

^2^Computational Biology and Clinical Informatics, Baker Heart and Diabetes Institute, Victoria, Australia

^3^School of Computing and Information Systems, University of Melbourne, Victoria, Australia

^4^Department of Biochemistry, University of Cambridge, Cambridge, UK


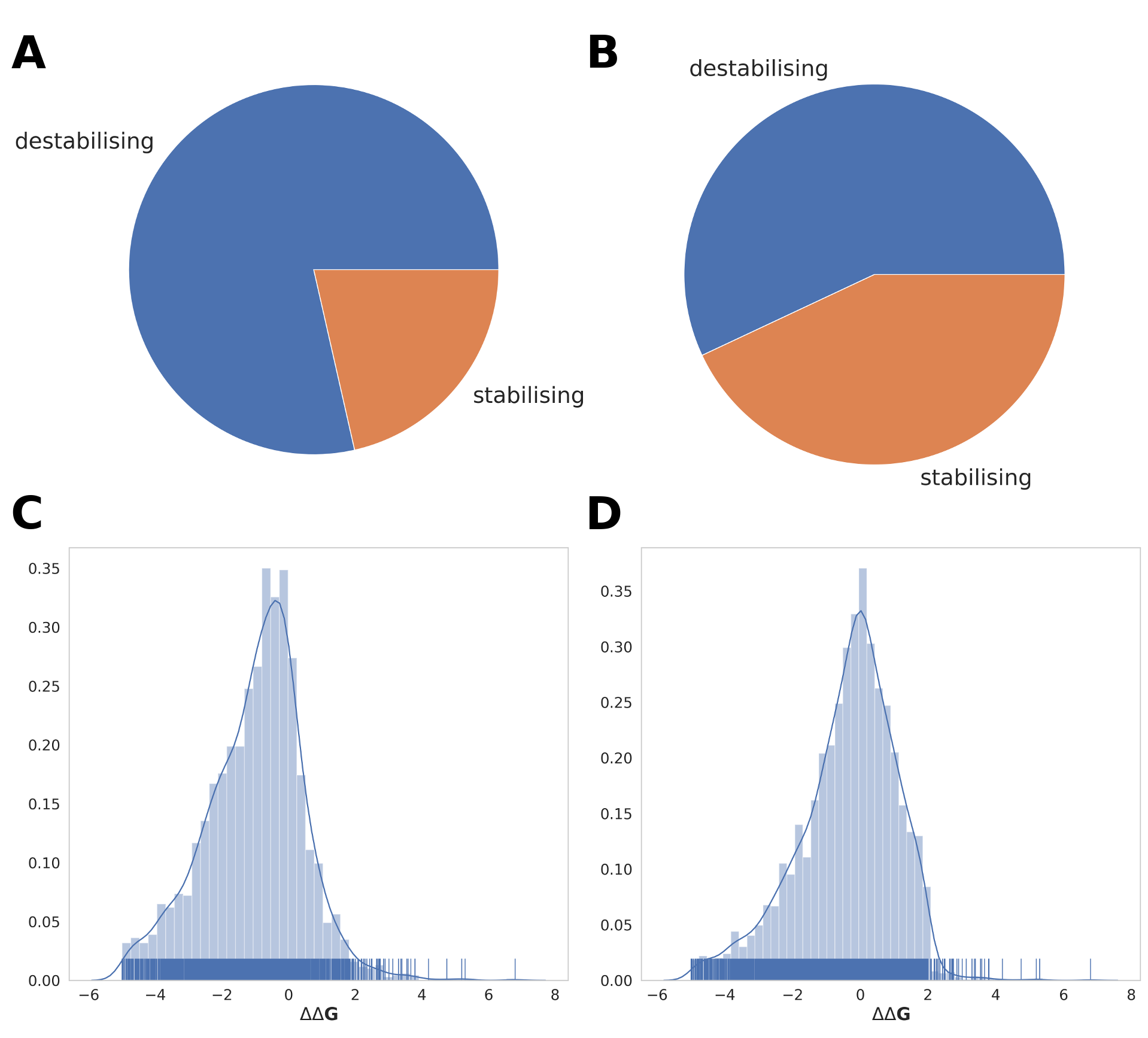


**Figure S1** - Proportion of stabilising and destabilising mutations on S2648 and ΔΔG distribution. A) highlights the unbalanced nature of the original dataset with a much greater number of destabilising mutations over the stabilising ones. B) depicts the proportions for each one of the classes after including the reverse mutations. C) and D) shows the distribution of ΔΔG values for S2648 and our final dataset used in this study.

**
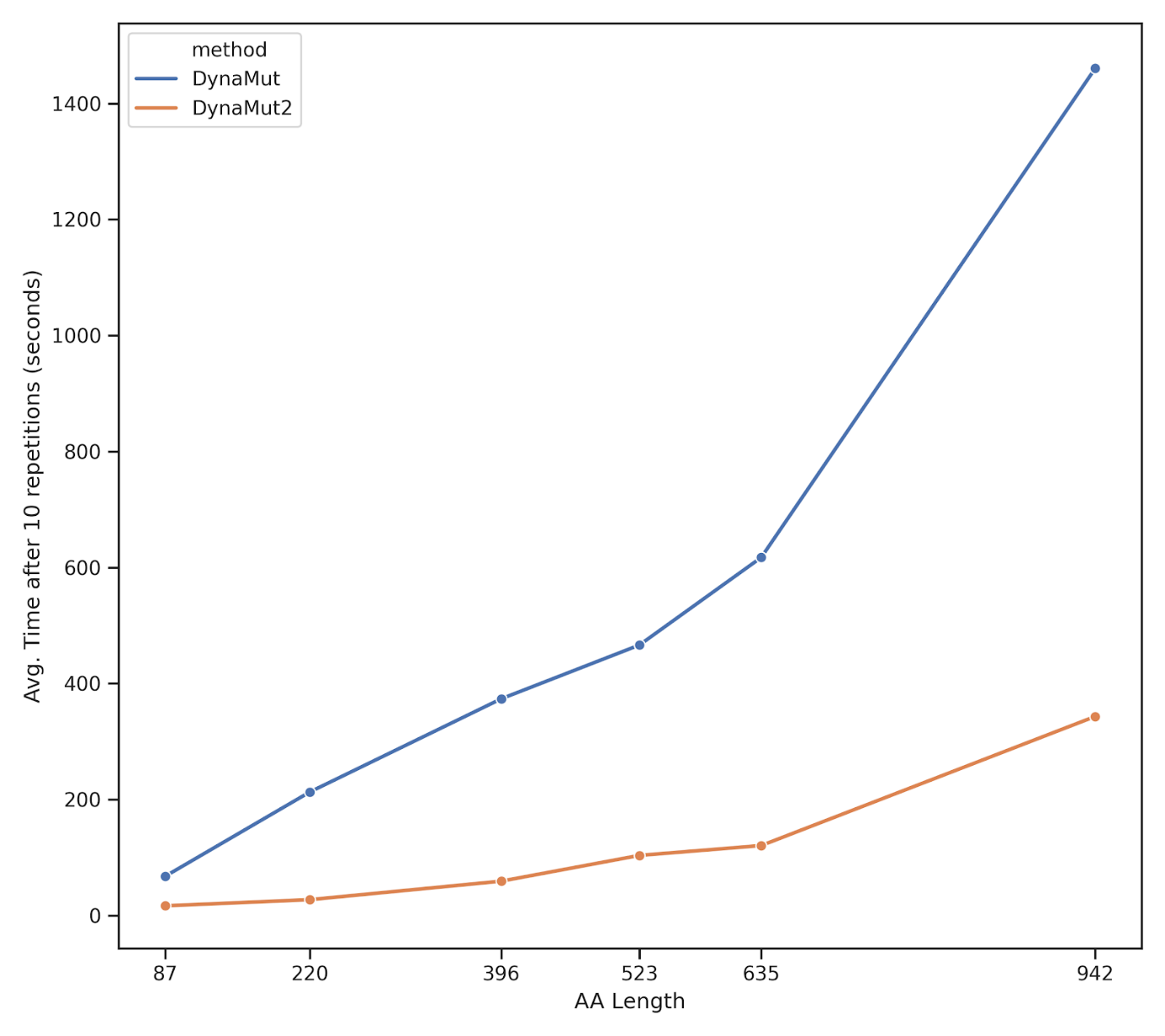
**

**Figure S2** - Processing time of DynaMut and DynaMut2 on structures of different sizes. Here we show average values of processing time (in seconds) for Dynamut (blue) and DynaMut2 (orange) after 10 repetitions across 6 different proteins. DynaMut2 has a much lower processing time than its previous implementation on all cases, including a decrease of more than 6 times on larger structures. Details on structures and mutations are shown in Table S13.


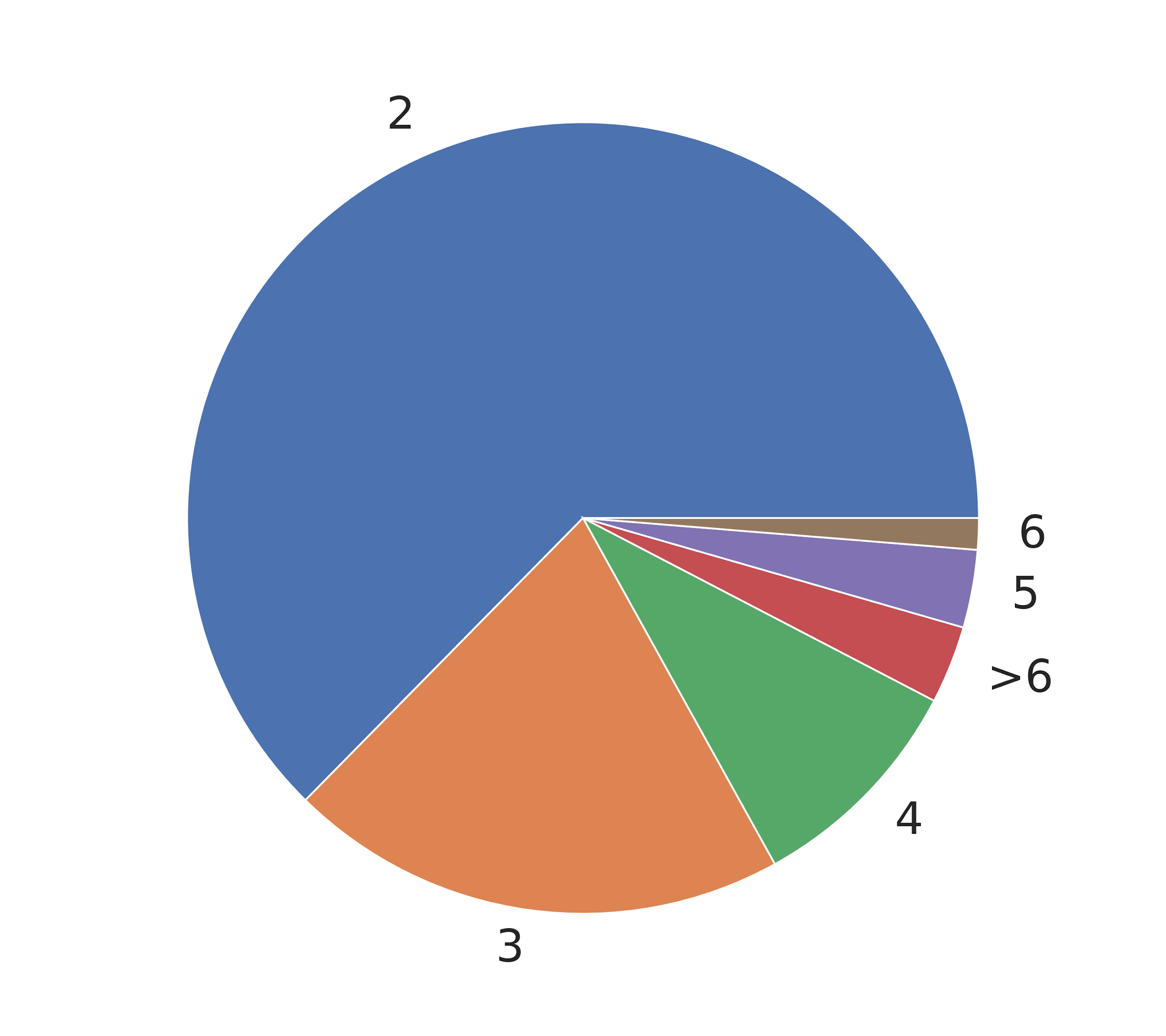


**Figure S3** - Distribution of number of point mutations from the original dataset extracted from Protherm. More than 80% of the entries in the dataset comprises double and triple mutants.


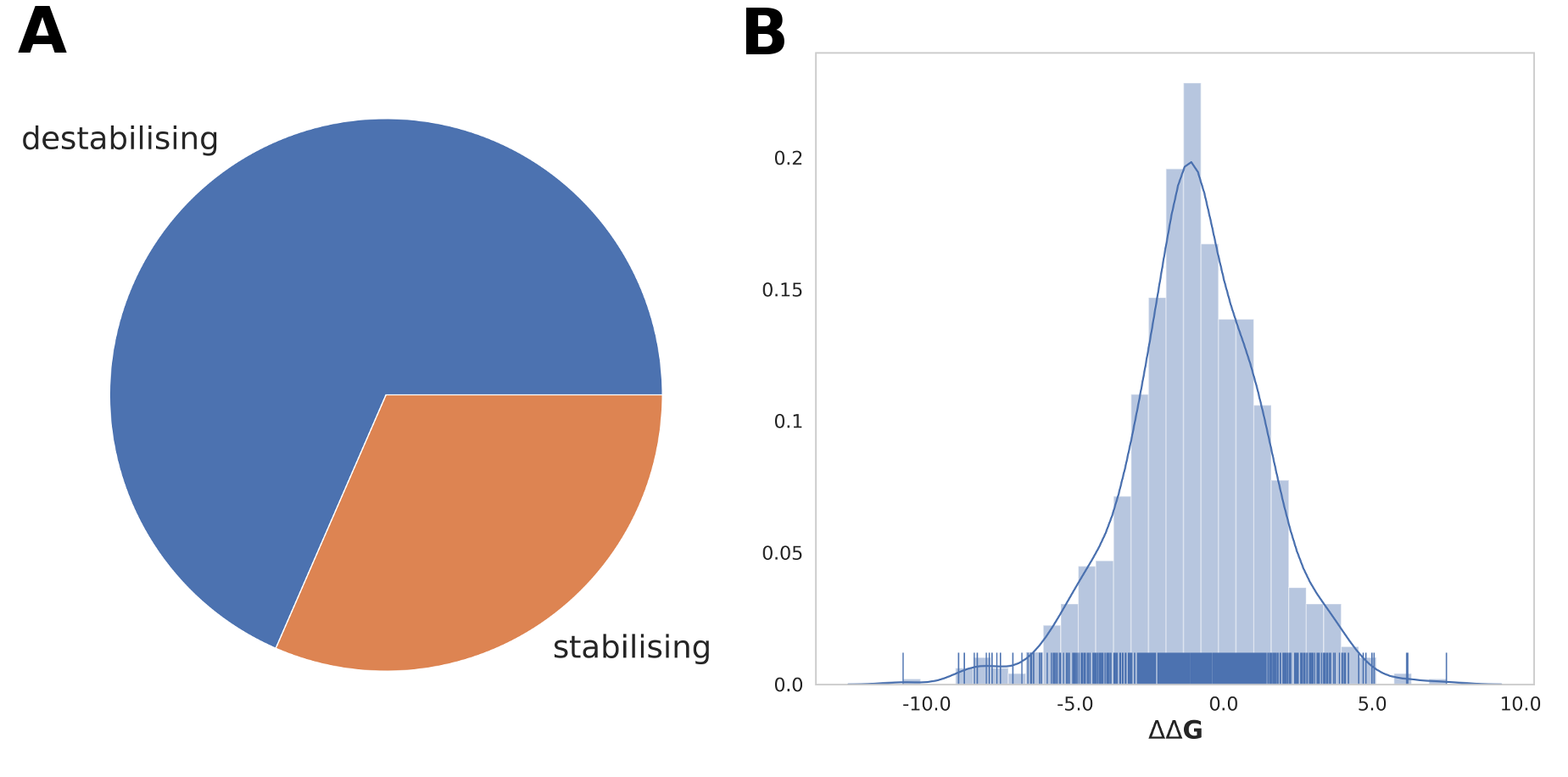


**Figure S4** - Distribution of ΔΔG values for our dataset of multiple mutations.

**Table S1** - Comparative performance on single-point mutation prediction on the test set S611 using rank correlation coefficients.

| Methods | Overall | | Stabilising | | Destabilising | |
| --- | --- | --- | --- | --- | --- | --- |
|  | Kendall | Spearman | Kendall | Spearman | Kendall | Spearman |
| **DynaMut2** | **0.42** | **0.58** | **0.22** | **0.27** | **0.39** | **0.56** |
| DynaMut | 0.27^*^ | 0.37^+^ | 0.11^*^ | 0.12^+^ | 0.36 | 0.52 |
| DUET | 0.25^*^ | 0.36^+^ | 0.01^*^ | -0.01^+^ | 0.35 | 0.50 |
| mCSM | 0.23^*^ | 0.33^+^ | 0.01^*^ | 0.01^+^ | 0.33 | 0.47 |
| SDM | 0.23^*^ | 0.32^+^ | 0.09^*^ | 0.13^+^ | 0.24^*^ | 0.35^+^ |
| ENCoM | -0.23^*^ | -0.33^+^ | -0.11^*^ | -0.16^+^ | -0.21^*^ | -0.32^+^ |
| Maestro | -0.15^*^ | -0.23^+^ | -0.0660^*^ | -0.09^+^ | -0.22^*^ | -0.33^+^ |
| I-Mutant | 0.15^*^ | 0.22^+^ | -0.07^*^ | -0.11^+^ | 0.31^*^ | 0.44^+^ |
| MUpro | 0.07^*^ | 0.11^+^ | -0.05^*^ | -0.07^+^ | 0.13^*^ | 0.21^+^ |

* p-value < 0.05 by transforming tau-to-r followed by Fisher r-to-z transformation
+ p-value < 0.05 by transforming rho-to-r followed by Fisher r-to-z transformation

**Table S2** - Comparative performance for single-point mutations on buried residues (RSA ≤ 30%) derived from the O2567 non-redundant to training sets for each model.

| Tool | Pearson | MAE |
| --- | --- | --- |
| **DynaMut2** | **0.41** | **1.42** |
| mCSM | 0.24* | 1.40 |
| FoldX | 0.21* | 2.09 |
| I-Mutant 3.0 | 0.14* | 1.37 |
| PoPMuSiC | 0.19* | 1.49 |
| SDM | 0.14* | 1.55 |
| Maestro | 0.25* | 1.41 |
| CUPSAT | 0.24* | 1.87 |
| Automute | 0.26* | 1.28 |

** p-value < 0.05 compared with DynaMut2 using Fisher r-to-z transformation*

**Table S3** - Comparative performance for single-point mutations on exposed residues (RSA > 30%) derived from the O2567 non-redundant to training sets for each model.

| Tool | Pearson | MAE |
| --- | --- | --- |
| **DynaMut2** | **0.27** | **1.42** |
| mCSM | 0.27 | 0.96 |
| FoldX | 0.16* | 1.41 |
| I-Mutant 3.0 | 0.15* | 0.86 |
| PoPMuSiC | 0.16* | 0.94 |
| SDM | 0.20 | 0.90 |
| Maestro | 0.37* | 0.83 |
| CUPSAT | 0.16* | 1.31 |
| Automute | 0.14* | 1.43 |

** p-value < 0.05 compared with DynaMut2 using Fisher r-to-z transformation*

**Table S4** - Comparative performance for single-point mutations on β-sheet structures according to CATH and derived from the O2567 non-redundant to training sets for each model.

| Tool | Pearson | MAE |
| --- | --- | --- |
| **DynaMut2** | **0.45** | **1.53** |
| mCSM | 0.21* | 1.66 |
| FoldX | 0.27* | 2.23 |
| I-Mutant 3.0 | 0.02* | 1.78 |
| PoPMuSiC | 0.24* | 1.66 |
| SDM | -0.05* | 1.89 |
| Maestro | 0.26* | 1.66 |
| CUPSAT | 0.31* | 2.05 |
| Automute | 0.08* | 1.51 |

** p-value < 0.05 compared with DynaMut2 using Fisher r-to-z transformation*

**Table S5** - Comparative performance for single-point mutations on α-helix and β-helix structures according to CATH and derived from the O2567 non-redundant to training sets for each model.

| Tool | Pearson | MAE |
| --- | --- | --- |
| **DynaMut2** | **0.37** | **0.99** |
| mCSM | 0.25* | 1.23 |
| FoldX | 0.25* | 1.65 |
| I-Mutant 3.0 | 0.24* | 0.89 |
| PoPMuSiC | 0.27* | 1.22 |
| SDM | 0.21* | 1.34 |
| Maestro | 0.32 | 1.22 |
| CUPSAT | 0.23* | 1.62 |
| Automute | 0.24* | 1.39 |

** p-value < 0.05 compared with DynaMut2 using Fisher r-to-z transformation*

**Table S6** - Comparative performance for single-point mutations on proteins with greater than 150 residues derived from the O2567 non-redundant to training sets for each model.

| Tool | Pearson | MAE |
| --- | --- | --- |
| **DynaMut2** | **0.43** | **1.47** |
| mCSM | 0.18* | 1.59 |
| FoldX | 0.21* | 1.98 |
| I-Mutant 3.0 | 0.11* | 1.48 |
| PoPMuSiC | 0.07* | 1.85 |
| SDM | 0.20* | 1.61 |
| Maestro | 0.28* | 1.54 |
| CUPSAT | 0.26* | 2.06 |
| Automute | 0.24* | 1.50 |

** p-value < 0.05 compared with DynaMut2 using Fisher r-to-z transformation*

**Table S7** - Comparative performance for single-point mutations on proteins with less than 150 residues derived from the O2567 non-redundant to training sets for each model.

| Tool | Pearson | MAE |
| --- | --- | --- |
| **DynaMut2** | **0.40** | **1.02** |
| mCSM | 0.27* | 0.94 |
| FoldX | 0.24* | 1.59 |
| I-Mutant 3.0 | 0.27* | 0.91 |
| PoPMuSiC | 0.37 | 0.90 |
| SDM | 0.23* | 0.99 |
| Maestro | 0.37 | 0.89 |
| CUPSAT | 0.18* | 1.32 |
| Automute | 0.22* | 1.30 |

** p-value < 0.05 compared with DynaMut2 using Fisher r-to-z transformation*

**Table S8** - Comparative performance for single-point mutations from large to small residues (in terms of volume) derived from the O2567 non-redundant to training sets for each model.

| Tool | Pearson | MAE |
| --- | --- | --- |
| **DynaMut2** | **0.37** | **1.66** |
| mCSM | 0.15* | 1.59 |
| FoldX | 0.21* | 1.89 |
| I-Mutant 3.0 | 0.12* | 1.63 |
| PoPMuSiC | 0.07* | 1.87 |
| SDM | 0.25* | 2.06 |
| Maestro | 0.23* | 1.67 |
| CUPSAT | 0.03* | 2.39 |
| Automute | 0.21* | 1.56 |

** p-value < 0.05 compared with DynaMut2 using Fisher r-to-z transformation*

**Table S9** - Comparative performance for single-point mutations from small to large residues (in terms of volume) derived from the O2567 non-redundant to training sets for each model.

| Tool | Pearson | MAE |
| --- | --- | --- |
| **DynaMut2** | **0.47** | **1.40** |
| mCSM | 0.09* | 1.78 |
| FoldX | 0.10* | 2.53 |
| I-Mutant 3.0 | -0.31* | 1.52 |
| PoPMuSiC | 0.11* | 1.74 |
| SDM | -0.11* | 1.72 |
| Maestro | 0.28* | 1.82 |
| CUPSAT | 0.43 | 1.81 |
| Automute | 0.41 | 1.22 |

** p-value < 0.05 compared with DynaMut2 using Fisher r-to-z transformation*

**Table S10** - Comparative performance for single-point mutations from and to residues with similar volumes derived from the O2567 non-redundant to training sets for each model.

| Tool | Pearson | MAE |
| --- | --- | --- |
| **DynaMut2** | **0.39** | **0.98** |
| mCSM | 0.35 | 0.96 |
| FoldX | 0.24* | 1.60 |
| I-Mutant 3.0 | 0.33 | 0.89 |
| PoPMuSiC | 0.38 | 0.91 |
| SDM | 0.32 | 0.97 |
| Maestro | 0.40 | 0.88 |
| CUPSAT | 0.32 | 1.36 |
| Automute | 0.23* | 1.31 |

** p-value < 0.05 compared with DynaMut2 using Fisher r-to-z transformation*

**Table S11** - Pearson Correlation coefficient for performance over blind test S173 of experimental ΔTm.

| **Method** | **1AQH** | **1H8V** | **1OSI** | **1XAS** | **2FJF** | **GK^a^** | **average** |
| --- | --- | --- | --- | --- | --- | --- | --- |
| DynaMut2 | -0.33 | 0.25 | 0.23 | 0.35 | 0.40 | 0.45 | 0.23 |
| DeepDDG | 0.69 | 0.16 | 0.25 | 0.56 | 0.76 | 0.50 | 0.49 |
| STRUM | 0.24 | 0.09 | 0.30 | 0.34 | 0.62 | 0.39 | 0.33 |
| SDM | -0.05 | 0.30 | 0.32 | 0.09 | 0.58 | 0.33 | 0.26 |
| DUET | -0.43 | 0.33 | 0.36 | 0.41 | 0.39 | 0.37 | 0.24 |
| mCSM | -0.55 | 0.22 | 0.32 | 0.33 | 0.34 | 0.34 | 0.17 |
| I-Mutant | -0.29 | 0.03 | 0.32 | 0.30 | 0.25 | 0.30 | 0.15 |
| MUpro | 0.31 | 0.12 | 0.18 | -0.47 | 0.30 | 0.33 | 0.13 |
| DynaMut | -0.63 | 0.21 | 0.23 | 0.12 | 0.29 | 0.33 | 0.09 |

*^a^ Guanylate kinase*

**Table S12** - NMA force field options available on DynaMut2.

| **Name** | **Description** |
| --- | --- |
| C-alpha (1) | Force field derived from fitting to the Amber94 all-atom potential |
| ANM (2) | Anisotropic Network Model uses a simplified spring force constant based on the pair-wise distance. |
| pfANM (3) | parameter-free Anisotropic Network Model is a variant from the ANM force field with interactions that fall off with the square of the distance. |
| REACH (4) | Realistic Extension Algorithm via Covariance Hessian is parameterized based on variance-covariance matrices obtained from MD simulations. |
| sdENM (5) | This force field employs residue specific spring force constants and it has been parameterized through a statistical analysis of 1500 NMR ensembles. |

**Table S13** - Summary of processing time for DynaMut and DynaMut2. Here we show average and standard deviation values after 10 repetitions for each mutation. Average values are shown with a confidence interval of 95%.

| PDB | Mutation | Chain | DynaMut (avg.) | DynaMut (std.) | **DynaMut2 (avg.)** | **DynaMut2 (std.)** |
| --- | --- | --- | --- | --- | --- | --- |
| 1A43 | G156A | A | 67.50 ± 0.67^*^ | 1.08 | **16.70 ± 1.17** | **1.89** |
| 1AKY | V8I | A | 212.90 ± 1.53^*^ | 2.47 | **22.00 ± 0.41** | **0.67** |
| 1AMQ | C270A | A | 373.60 ± 2.67^*^ | 4.33 | **43.25 ± 0.40** | **0.63** |
| 1AON | T516V | A | 466.30 ± 5.27^*^ | 8.50 | **81.40  ± 2.86** | **4.62** |
| 2ZT8 | T526V | A | 617.30 ± 2.50^*^ | 4.03 | **112.10  ± 1.28** | **2.07** |
| 6M71 | Y884K | A | 1460.50 ± 6.02^*^ | 9.71 | **231.90 ± 3.42** | **5.53** |

* p-value < 0.05 when evaluating average values with  t-Test

**REFERENCES**

1. Hayward S, Kitao A, Go N (1995) Harmonicity and anharmonicity in protein dynamics: a normal mode analysis and principal component analysis. Proteins 23:177-186. PMID: 8592699 {Medline}

2. Atilgan AR, Durell SR, Jernigan RL, Demirel MC, Keskin O, Bahar I (2001) Anisotropy of fluctuation dynamics of proteins with an elastic network model. Biophys J 80:505-515. PMID: 11159421 {Medline}

3. Yang L, Song G, Jernigan RL (2009) Protein elastic network models and the ranges of cooperativity. Proc Natl Acad Sci U S A 106:12347-12352. PMID: 19617554 {Medline}

4. Moritsugu K, Smith JC (2007) Coarse-grained biomolecular simulation with REACH: realistic extension algorithm via covariance Hessian. Biophys J 93:3460-3469. PMID: 17693469 {Medline}

5. Dehouck Y, Mikhailov AS (2013) Effective harmonic potentials: insights into the internal cooperativity and sequence-specificity of protein dynamics. PLoS Comput Biol 9:e1003209. PMID: 24009495 {Medline}
